# The Value of Children's Voices for a Video Game Development in the Context of Type 1 Diabetes: Focus Group Study

**DOI:** 10.2196/diabetes.7652

**Published:** 2017-07-19

**Authors:** Valéria de Cássia Sparapani, Sidney Fels, Lucila Castanheira Nascimento

**Affiliations:** 1 Ribeirao Preto College of Nursing, World Health Organization Collaborating Centre for Nursing Research Development Department of Maternal-Infant and Public Health Nursing University of São Paulo Ribeirão Preto Brazil; 2 Electrical and Computer Engineering Department of Electrical and Computer Engineering The University of British Columbia Vancouver, BC Canada

**Keywords:** type 1 diabetes mellitus, video games, qualitative research, pediatric nursing, serious games, self-management

## Abstract

**Background:**

Children with type 1 diabetes mellitus (T1DM) face daily challenges performing self-care tasks, controlling symptoms, and dealing with psychosocial issues. The use of video games to improve health is a successful support for persons with chronic diseases, promoting adequate self-management through simulations of real life. Involving future users in the development of games is essential to generating innovative, creative, and effective programs.

**Objective:**

Our goal is to identify what children with T1DM need to know about their disease and their self-care tasks as well as their preferences in video games.

**Methods:**

Children with T1DM provided input about their learning needs, self-care tasks, and preferences in video games. Three categories were identified through qualitative content analysis: dealing with emotions and knowledge, practical skills and awareness, and game preferences.

**Results:**

Children expressed concerns about the difficulties of self-care, lack of knowledge about diabetes, and lack of awareness about the consequences of behaviors related to self-care, which contribute to inappropriate behaviors and significantly impact self-management of their disease. They expressed enthusiasm for a video game for children with diabetes that considered their needs and preferences.

**Conclusions:**

Findings support the potential benefits when children’s input is considered in game design. Consideration of customer needs and preferences is a powerful resource in the development of video games with enhanced learning experience.

## Introduction

Type 1 diabetes mellitus (T1DM) is one of the chronic diseases that most affect children and adolescents [1-3]. Adherence to a treatment regimen is a key component in its management; however, poor management is a common problem among children with diabetes due to lack of knowledge that leads to inadequate behaviors and undeveloped skills [1]. The lack of disease understanding [4] and challenges of being a child with T1DM [5,6] may be associated with treatment nonadherence. This scenario demands educational interventions that take into account individuals and consider their clinical care routine and psychosocial needs. These interventions must engage parents and health professionals and use psychoeducational principles and behavioral procedures [7,8] and the design of new technologies [9].

Video games have been cited in the literature as tools that capture children’s attention and promote understanding and learning about their condition. Some studies report the positive effects of using video games on health determinants [10] and clinical outcomes [1]. These games use strategies that can motivate positive behavioral changes, thereby assisting in disease self-management and health promotion [3,11]. Video games aimed at children with T1DM began to be developed in the 1990s [13]. In general, studies have shown positive results from the use of these games, such as a reduction in the number of urgent care visits, improvement in self-efficacy and self-care, and improved communication about the disease among children, parents, and friends [12-16]. The best results are achieved when there is a familiarity between the player and the main character in the game, whether in the physical appearance or similar clinical conditions [15,16]. Conversely, some studies specifically aimed at children and adolescents with diabetes by using fantastical characters and themes such as elephants, card games, and plane trips [17,18]. Prompted by the potential of these tools for health improvement, our long-term objective is to develop a video game for children with T1DM that focuses on knowledge about the disease and self-care tasks.

Video games designed for children and adolescents with T1DM and type 2 diabetes mellitus (T2DM) have been the focus of literature reviews [12,14], research interventions [13,14,19], and studies on the use of conceptual frameworks for game development in this field [8,20,21]. However, few studies considered the needs and preferences of the target population during videogame development.

A recent study [22] investigated the contribution of children’s experiences about diabetes self-care during the early stages of development of a video game about insulin injections. Considering the input from future users is an important step in the development of successful approaches for interactive technologies [22,23]. The following components should be considered: cognitive aspects; language abilities; literacy level [24]; particularities concerning age and culture; and needs, preferences, and experiences [3].

Although barriers that may interfere in the self-management of diabetes are well recognized in the literature, the reasons for nonadherence to treatment are linked to differences in each individual and group and these must be investigated [25]. Although video games can provide health benefits, they cannot achieve their goals unless the profile, needs, and preferences of children are considered.

Therefore, in order to improve the design of a future video game, we conducted a qualitative study that included the following research questions: What are the main learning needs related to understanding the disease and self-care tasks from the perspective of children with T1DM? How should the video game be designed to appeal to the children with TIDM? Our goal is to identify what children with T1DM need to know about their disease and their self-care tasks as well as their preferences in video games.

## Methods

### Participants

A total of 19 children, 5 boys and 14 girls with a mean age of 9.8 (SD 1.8) years and the mean time since diagnosis of 3.5 years, participated in the study. The mean hemoglobin A_1c_ value was 9.8% in the last year of follow-up. Of the 19 children, 15 (79%) were using regular and neutral protamine Hagedorn insulin, 3 (14%) were using rapid and long-acting analogs, and 1 child (5%) used an insulin pump. All children were living in urban areas and attending school. Children were recruited at the Endocrinology and Childhood Diabetes Outpatient Clinic from educational group meetings. The clinic’s multidisciplinary team provides diabetes education during weekly group meetings. For the past 5 years, these group meetings have been led by one the authors of the present study (V Sparapani), who is a nurse with experience in research, children, and parents.

The eligibility criteria included children (boys and girls) aged 7 to 12 years with a diagnosis of T1DM, regardless of the time of diagnosis. The exclusion criterion was any form of developmental delay that could interfere with the data collection strategy. The presence of developmental delay was evaluated using information provided by the health team and medical records. The lead nurse explained the activity to the children and parents, who were participants in educational group meetings, and presented the study goals, potential risks and benefits, and their rights to withdraw from participating at any time. The researcher allowed parents some time to freely consent to their children’s participation in the activity. The study was approved by the institutional review board of the university and hospital. All parents of study participants provided written informed consent, and children also gave their assent to participate.

### Focus Groups

The focus groups technique, a method which promotes an environment of interaction and discussion on a given topic among participants [8], has been widely used in research [11,17,20]. This technique is also used in the development of interactive technologies because it considers the user involvement in the process of technology development from conception to final evaluation. We adopted a user-centered design (UCD) approach [3,8,24,26] for this study, an approach requiring utilization of focus groups.

Data were collected from December 2012 to May 2013. The study included 6 40- to 60-minute focus group meetings consisting of 4 to 6 children per group, 6 groups in total. Four children participated twice, totaling 23 participations. These double participations resulted from the number of follow-up consultations the children had at the outpatient clinic and their willingness to participate, not configuring restrictions or exclusions from the focus groups. Focus groups were moderated by the main author of this study; a research assistant took notes from verbal and nonverbal communication. The meetings were held in a private room in the hospital concurrent to the educational meetings conducted with the children’s parents. Children were aware that their parents were nearby.

According to recommendations in the literature [8], the moderator tried to create a comfortable and trustworthy atmosphere to motivate participants to share their experiences and feelings. The children were invited to sit in a circle on the floor, received name tags, and were introduced to each other to break the ice before the session started.

Because young children demonstrate differences in comprehension levels, abilities, sensitivities, abstraction capabilities, styles [8,27,28], and capabilities with video games of different complexity levels, children were assigned to 1 of 2 focus groups according to their age (7 to 9 and 10 to 12 years old). This strategy enabled us to identify age-appropriate tasks for video game development. The researcher discussed the focus group and study goals with the children and described what was expected of them. The groups were audiotaped with the children’s and parents’ permission. The following open-ended questions guided the activity: Why is insulin necessary? What is the most difficult task in the diabetes treatment? Why is that task difficult? What kind of video game do you like most? What would you like to see in a video game for children with diabetes? Every child had the opportunity to answer to every question. The researchers finalized the data collection through the focus group sessions when the data set from these focus groups was sufficient to reach the study’s objectives.

### Data Analysis

The focus group data were analyzed following deductive and inductive content analysis guidelines [29,30]. The resulting information was organized into 3 phases: preparation, organization, and reporting. During the preparation phase, data were fully transcribed and read several times until the researcher was acquainted with the contents. In the organization phase, the researcher made careful notes, defined text headings, and elaborated categories and subcategories based on data analysis [29,30]. The children’s excerpts were selected to support the discussions in the reporting phase [29,30].

## Results

Qualitative analysis identified 3 main categories representing learning needs related to knowledge about the disease, self-care tasks, and the children’s video game preferences.

### Dealing With Emotions

The children’s statements brought up emotions that interfere with the proper performance of self-care tasks. Fear *,* insecurity, and pain were emotions linked to insulin injection.

The insulin needle is tiny, but I’m scared of it.

Girl, 11 years

*When I take the insulin syringe, I have a bad feeling! It’s not really that I’m scared, I’m scared to make a mistake somewhere in my body, you know? [Boy, 11 years]*

I don’t shoot it because it hurts.

Girl, 8 years

The children reported anger about having to self-monitor blood glucose (SMBG) several times per day and therefore did not monitor appropriately. One child said that she does not like to perform this task, fails to execute it, and chooses when she wants to perform it.

I don’t like doing the test. I switch the days that I do the test. If I do the test today at dinner, tomorrow, I don’t do it... It’s bad, and I also forget to do it. I don’t like doing the test

Girl, 13 years

Learning how to deal with desire was also an identified need. The children reported uncontrollable emotions related to consuming foods in large quantities, particularly sweets.

I am nibbling food all the time! I can’t control myself. I will ask the doctor to be hospitalized. If I am at the hospital, I can comply with the diet correctly.

Girl, 8 years

Children were also demotivated regarding healthy eating habits. Some participants do not feel motivated to eat vegetables because they do not like them. Conversely, other children would like to comply with a proper diet; however, they find themselves demotivated due to the lack of support from parents. One child shared that she asked her mother to buy fruits and vegetables because they are not frequently available in her home.

I probably don’t eat vegetables because of my mother. She doesn’t make salads frequently... When I ask, she says, ‘Tomorrow I buy it’ or ‘Wait’ or ‘I’ll go to the grocery store later.’

Girl, 12 years

Children also have no incentives to practice physical activities. They do not accomplish this self-care task and provided several reasons for not doing it.

I don’t have a bicycle. There’s no place for walking or hiking. There is a park, but with no covered area. There are rocks all over the floor...and a lake around it. I like it, but it’s bad to go there alone. My mom doesn’t like it.

Girl, 8 years

### Knowledge, Practical Skills, and Awareness

Learning needs related to understanding the disease and self-care, practical skills required for self-care, and awareness about the consequences of favorable or unfavorable behaviors related to self-care were identified. One child demonstrated her lack of knowledge about the causes and deficiencies in T1DM.

I think I have diabetes because I had too much sugar in the pancreas. It couldn't take it, and it stopped working.

Girl, 12 years

The children demonstrated learning needs regarding the function of insulin. According to the statements, the insulin has the role to “kill,” “break,” or “dissolve” the sugar present in the blood.

My body has no insulin to kill sugar. Insulin kills the sugar...

Girl, 8 years

The children demonstrated not knowing the food groups and different energy contents. A child talked about a hypoglycemia episode and questioned the moderator about what had caused the glycemic level drop since she had eaten at a barbecue place. According to the girl, she did not understand the explanation given by a relative, who said that she had to ingest other kinds of food and not just meat.

I was at a barbecue. When I got back home, I was feeling sick. I did the blood glucose test, and it was 25. I asked my aunt why it was 25 as I had eaten, and she said, ‘It is the roast beef...’ I didn't quite understand, but she said that when we go to a barbecue and only eat meat, we also have to eat other things. I didn't quite understand.

Girl, 12 years

Moderator: *Does meat have a lot or little energy?*

I think that it has a lot!

Girl, 12 years

Moderator: *Is meat in the potato group?*

No.

Girl, 12 years

Moderator: *To which food group does meat belong?*

Proteins.

Girl, 12 years

Moderator: *And does meat have more or less carbohydrate?*

Less. Is it because of this?

Girl, 12 years

The dialogue demonstrated the lack of practical skills required to carry out effective self-care. The children reported inappropriate techniques and doubts regarding the delimitation of injection sites and what to do with air bubbles in the syringe or in the insulin pen.

Can I apply insulin here [showing the inner thigh]? I apply here! I became accustomed to doing it.

Girl, 12 years

The deficiency in practical skills related to carbohydrate counting was observed in several children, a potential reason for nonadherence to this task.

My mother and I weren't able to do the carbohydrate counting. We thought it very difficult. Sometimes my mother would go to work, and I couldn't make the counting alone.

Girl, 11 years

Some children reported that they use the same SMBG lancet more than once because they do not have the practical skills to change the needle in the lancing device and usually puncture the same body site.

*I change the lancet device only after a long time in use because then it does not stick anymore. [Girl, 11 years]*

I find it hard to change the needle in the lancing device. It seems that the needle will fall, and I'm afraid to stick my finger on it.

Girl, 8 years

The participants showed injuries on their fingers indicating that they use only the middle finger and index finger to perform the test. The children’s lack of awareness was evident about the consequences of favorable or unfavorable behaviors to self-care. Many participants acknowledged that they had lipodystrophy because they do not use all available body sites for insulin application. The moderator asked, “And do you know why this lump is over there?” The child answered, “Because I apply here a lot.”

Other children mentioned that although they know about the standard diet that would help them maintain adequate blood glucose levels, they do not follow it.

The doctor explained to me that when there are pasta and rice I can only choose one to eat. If I want to choose both, I can get half portion of one and a half portion of the other. I don't want to! I want everything, or I don't want any.

Girl, 12 years

The participants do not perform correct monitoring records using excuses such as “I forgot.” Many of the children assumed they do not perform the SBMG when there is some possibility of nonideal results.

Once I woke up at an early hour and ate half of the chocolate bar from my sister... Then, I didn’t even measure the blood glucose in the morning.

Girl, 9 years

The participants reported a significant number of hypoglycemia episodes, especially during physical activity. They demonstrated a lack of awareness regarding the consequences of eating chocolates and other sweets during these hypoglycemia episodes. A child told us about the food she uses for treating hypoglycemia episodes.

Sweets. My mom gives me candy, cookies, and chocolate.

Girl, 9 years

### Game Preferences

The children provided opinions about the video games they like to play. They also presented ideas about what a video game for children with diabetes should be like. Many participants expressed an interest in seeing what happens inside their bodies as the result of diabetes through a video game.

It could be a game in which the character had a spaceship. He could enter through our mouths and go inside of us. He would go through everything we have inside our body, and thus, the game stages would unfold.

Boy, 10 years

The character should answer three questions to pass through stages. These questions would be related to what he saw inside the body...

Girl, 12 years

The children would like to learn how to better control their diabetes, mostly about what they can or cannot eat.

I would like to play an eating game... A game in which we could choose what we should eat. The amount of sugar and carbohydrates. I saw a video game equal to that at the mall. I played it.

Boy, 10 years

The participants would like to see a task about carbohydrate counting in the video game for diabetes. They want the opportunity to learn how to perform this task properly.

Somewhere in the game, it could have a restaurant that you entered or you’d have to click on. In this location, you could have an explanation on the easiest way to make the carbohydrate counting. I think that I could learn in this way.

Girl, 11 years

According to their preferences, they want to learn how to perform insulin injections properly and how to train on other materials.

I would like to see the game character applying insulin in a doll.

Girl, 10 years

The children said that physical activities, such as swimming or cycling, could help them understand diabetes.

The game could help us counting energy levels. Each movement burns a little amount of energy and the game could go on explaining this to us..

Girl, 13 years

The participants also contributed with ideas for components such as the game environment, scoring system, and characters. They emphasized their preferences for adventure games.

It could be an adventure game. A space travel.

Girl, 11 years

In another world... We want to go to new places... A park, a zoo, a forest! ... But I would like the game to talk only about diabetes.

Girl, 10 years

The researcher also asked the children how they could win in the game or collect points during the story.

If we make the right choices, we'll be getting stronger.

Boy, 10 years

According to the children’s preferences, they would like to participate interactively in the construction of characters, choosing their physical form and clothes.

We could create the characters! We could choose the gender and the color of their eyes, hair, and their clothes and shoes.

Girl, 12 years

Yet, they would like to have someone to play the game with them:

We want friends.

Girl, 12 years

## Discussion

### Principal Findings

Accomplishing diabetes self-management demands knowledge about the disease and fulfillment of a care plan, which is not easy for any person and especially difficult for children because of their developmental stage and maturity [31,32]. The analysis of statements enabled us to identify gaps in the participants’ knowledge about T1DM and reasons why they should perform proper self-care. In addition, empirical data show emotions that contribute to the understanding of behaviors of nonadherence to treatment or failure to perform self-care tasks. Our findings corroborate previous studies that present fear and anxiety associated with needles [33], depression, and difficulty controlling desires related to nutritional therapy [5,33]. Some studies discuss the importance of knowledge about the disease to the appropriate performance of self-management tasks [34,35], while other studies describe the difficulties related to practical skills for self-care and the lack of awareness of these difficulties [36].

Parents’ attitudes considerably influence their child’s behavior, which shows the importance of parental encouragement and support in promoting appropriate self-care, such an appropriate diet [36].

This data analysis mirrors aspects already presented in the literature, demonstrating interactions between emotions, knowledge, difficulties in practical skills for self-care, and lack of awareness towards these difficulties. These interactions have a significant influence on diabetes management. The discussion that we propose has not been presented in previous studies or in studies about using video games to learn about T1DM.

In this study, participants demonstrated insecurities regarding insulin injections, which are intensified by their fear of needles. They do not feel safe performing the technique, especially in handling needles, because they fear damaging their bodies. Some of their deficiencies in these practical skills reflect doubts regarding appropriate injection sites and removal of bubbles inside the syringe, which increase the children’s insecurity about self-application.

The children who participated in this study revealed that they avoid performing insulin injection in the abdomen or upper buttocks because they are afraid of feeling pain. The rotation of sites chosen for insulin injection is strongly recommended to prevent lipohypertrophy [37], which was a complication observed in this study. Lipohypertrophy is the result of fat accumulations in the subcutaneous tissue where insulin is applied due to multiple injections in the same site [38]. The observation of injections in lipohypertrophy sites demonstrates the children’s lack of awareness about possible complications such as reduction in insulin absorption [38]. Although some children demonstrated awareness of some complications, their lack of knowledge about the function of insulin generated doubts as to whether or not the consequences of this action are adequately understood. The fear of insulin injections is associated with many complications such as poor glycemic control, clinical complications, and psychological comorbidity [39].

These data demonstrate the need for children with T1DM to learn how to deal with their emotions and to acquire practical skills related to insulin injections. Fear and pain are themes of studies that discuss the need to address these emotions in interventions with these children [39,40]. A video game called *Koodak-e-Tavana* was designed to teach children about diabetes and insulin injections and aimed at reducing fear and anxiety [22]; this study is one of the few involving children in the first step of the process that identifies needs and tasks which should be included in the game [22]. Insecurity, fear, and pain are not topics commonly discussed in video games developed for children with T1DM. However, creative and innovative approaches such as video games may be able to promote a simulated environment that involves the children and enables them to learn how to deal with these emotions. The fact that children were motivated to learn about their disease and how to manage it was evident when they wished for a game that showed them how to inject insulin and allowed them to practice injecting insulin in a doll. These tools can increase awareness about complications and improve practical skills for proper insulin injection. Behavioral changes can contribute to increased adherence to insulin therapy.

Children participating in this study stated that the recommended number of SMBG tests per day caused anger and that the use of a new lancet and new pricking site was painful: they keep using the same lancet or pricking the same finger for several days to avoid pain. According to Floch et al [41], the reuse of lancets and pricking sites demonstrates a deficiency in the children’s practical skill for this task. That study concluded that the pain and cutaneous finger injuries on pricking sites are related to frequently pricking the same sites, corroborating our results. These data indicate learning needs about facing pain and anger to improve the performance of these practical skills.

Similarly, blood glucose level evidenced by SMBG is another reason why the children say this is a bad task, because the results can reveal their nonadherence to treatment. The children’s lack of understanding of the importance of this task is evident when they report their failure to monitor their glucose levels as needed. SBMG is an instrument for patients, parents, and health staff that provides for improved glycemic control [42].

There are few studies in the literature that discuss the use of video game applications aimed at motivating children to perform the SBMG in detail [17]. One study encouraged 4 24-year-old patients with T1DM to perform the SMBG and transfer the results to the game system. The game gives players the opportunity to earn reward points based on blood glucose values. However, the study does not show if patients have increased the amount of tests performed [19]. Topics such as coping with pain and how to deal with emotions such as anger and awareness about the SMBG were not covered in that study [19] or in other published studies. A video game that considers these learning needs could be very useful in providing information and encouraging the appropriate performance of SMBG by children, which, consequently, would improve T1DM self-management.

The children’s awareness of the role of SMBG can be beneficial to the understanding of the influence of food on glycemic control, allowing them to make safer food choices. Learning about food groups and their energy loads and impact on glycemic control can support children in using practical skills related to nutritional therapy, such as planning meals. Carbohydrate counting was another learning need identified in this study. Carbohydrate counting is a technique for planning meals that aims to improve glycemic control and allows flexibility in food choices [43]. Children participating in this study stated that they would like to play a game that not only helps them to better select food choices but also provides an easier understanding about carbohydrate counting.

Dealing with the desire to consume sweets and other foods is another learning need identified in the children’s statements. Our results are similar to other findings [5,33,44] showing that uncontrolled desire and anxiety related to nutritional therapy are self-management barriers. There are few game strategies that address coping with these emotions [22]. *Captain Novolin*, a video game released in 1992, shows the main character fighting evil doughnuts, milkshakes, sodas, and other sweets to keep normal glucose levels [14]. A video game that promotes awareness about the effects of high consumption of sugars and other foods—presenting routines with parents and friends eating together and offering healthy food options [43]—can promote better results for coping with desires and anxieties related to food intake.

The participants also demonstrated demotivation regarding the consumption of healthy foods. The lack of parents’ incentives was demonstrated as a variable influencing this demotivation. A study by Baranowski et al [45] evaluates the influence of fruit and vegetable intake in the life of a child, the availability and accessibility of these foods at home, the role of parents as role models, and the purchase of these foods as impact factors. A video game developed to change the eating behavior of children by increasing fruit and vegetable intake achieved positive results; a game that promotes training parents to increase vegetable consumption in their preschool children is also cited in the literature [46]. An environment designed to explore the positive involvement of parents and friends in children’s daily routine can increase the frequency of healthy choices, helping them to deal with anxieties, desires, and demotivation that prevent adherence to the nutritional therapy.

The lack of motivation is also present in the practice of physical activities. Children are less physically active than recommended and provided several excuses [47]. However, the children mentioned preferences for an adventure video game, located in forests or in a zoo and requiring several exercises. Encouragement toward increasing physical activity and reducing sedentary behaviors is a usual theme in video games designed to reduce risks of obesity and T2DM [8,26]. Studies demonstrate that the major barrier to participating in physical activities is the child’s fear of hypoglycemia [31]. However, this issue was not identified in the dialogs from the children in our study who demonstrated inappropriate management of hypoglycemia episodes by eating foods with high amounts of fats such as chocolates or ice cream. This fact may be linked to the children’s desire for candy consumption. Fat consumption is not considered effective to treat hypoglycemia because it can slow down the absorption of carbohydrates [48], another learning need identified in this study.

The children expressed a desire to learn about energy expenditure during exercises in the video game. A video game script specifically designed for children with T1DM might work as an excellent strategy to motivate physical activity. It can lead to the development of a taste for exercising and promote understanding of the relationships between food, physical activity, and insulin in the control of diabetes [47]. Thus, the participation of family members and friends in playing the game can influence the children’s decision about becoming more active [8]. According to Brennan and Fink [49], family, friends, and people from other social relationships can have an influence on the children’s decisions about incorporating or avoiding certain behaviors.

The lack of knowledge about diabetes demonstrated by the participating children was related to insulin function, causes of diabetes, and the role of foods in the body. An increase in understanding of the disease has been shown in studies that used video games with goals of changing health behaviors through the use of educational interventions [17,50,51]. In this study, the children’s suggestions indicate their desire to experience what happens inside the body after insulin injections, meals, or physical activity through a video game. However, there are no reports in the literature about video games on the theme of understanding T1DM. This design strategy could be an innovative method of enabling children with T1DM to understand the disease in an easier manner and achieve the knowledge required for its management.

The implications for the development of a video game are related to valuing the participation of the target population in this crucial stage. The study included children who were participating in a diabetes education group. Perhaps if we had included children who had not participated in these educational groups, we could identify different needs and preferences.

### Conclusions

The findings of this study corroborate the importance of involving the children in the design of a video game. The analysis of the children’s experiences and ideas showed potential interactions between emotions, knowledge about the disease and self-care, difficulties in practical skills for self-care, and lack of awareness toward these difficulties.

The children’s input demonstrated the significant impact of identifying learning needs in diabetes self-management to develop improved learning experiences. Our future studies will focus on health behavior theories and behavioral determinants and their influence on the learning needs identified in this study to guide our long-term intervention goal, which is the development of a video game for children with T1DM.

## Abbreviations

SMBG: self-monitor blood glucose
T1DM: type 1 diabetes mellitus
T2DM: type 2 diabetes mellitus
UCD: user-centered design

## References

[1] International Diabetes Foundation Global IDF/ISPAD guidelines for diabetes in childhood and adolescence.

[2] DIAMOND Project Group (2006). Incidence and trends of childhood type 1 diabetes worldwide 1990-1999. Diabet Med.

[3] American Diabetes Association (2014). Standards of medical care in diabetes—2014. Diabetes Care.

[4] National Institute for Clinical Excellence Type 1 diabetes: diagnosis and management of type 1 diabetes in children, young people and adults.

[5] Delamater AM (2009). Psychological care of children and adolescents with diabetes. Pediatr Diabetes.

[6] Sparapani VC, Jacob E, Nascimento LC (2015). What is it like to be a child with type 1 diabetes mellitus?. Pediatr Nurs.

[7] Lange K, Swift P, Pańkowska E, Danne T, International Society for Pediatric and Adolescent Diabetes (2014). ISPAD Clinical Practice Consensus Guidelines 2014. Diabetes education in children and adolescents. Pediatr Diabetes.

[8] Thompson D, Baranowski T, Buday R, Baranowski J, Thompson V, Jago R, Griffith MJ (2010). Serious video games for health: how behavioral science guided the development of a serious video game. Simul Gaming.

[9] Hayes B, Aspray W (2010). Health Informatics: A Patient-Centered Approach to Diabetes.

[10] DeSmet A, Van Ryckeghem D, Compernolle S, Baranowski T, Thompson D, Crombez G, Poels K, Van Lippevelde W, Bastiaensens s, Van Cleemput K, Vandebosh H, Bourdeaudhuij I (2014). A meta-analysis of serious digital games for healthy lifestyle promotion. Prev Med.

[11] Street Jr RL, Rimal RN (1997). Health Promotion and Interactive Technology: Theoretical Applications and Future Directions.

[12] DeShazo J, Harris L, Pratt W (2010). Effective intervention or child's play? A review of video games for diabetes education. Diabetes Technol Ther.

[13] Brown SJ, Lieberman DA, Germeny BA, Fan YC, Wilson DM, Pasta DJ (1997). Educational video game for juvenile diabetes: results of a controlled trial. Med Inform (Lond).

[14] Lieberman DA (2012). Video games for diabetes self-management: examples and design strategies. J Diabetes Sci Technol.

[15] Fuchslocher A, Niesenhaus J, Krämer N (2011). Serious games for health: an empirical study of the game “Balance” for teenagers with diabetes mellitus. Entertain Comput.

[16] Theng Y, Lee JWY, Patinadan PV, Foo SSB (2015). The use of videogames, gamification, and virtual environments in the self-management of diabetes: a systematic review of evidence. Games Health J.

[17] Lieberman DA (2001). Management of chronic pediatric diseases with interactive health games: theory and research findings. J Ambul Care Manage.

[18] Martin C, Liveley KWK, Whitehead K (2009). A health education group intervention for children with type 1 diabetes. J Diabetes Nurs.

[19] Klingensmith G, Aisenberg J, Kaufman F, Halvorson M, Cruz E, Riordan ME, Varma C, Pardo S, Viggiani MT, Wallace JF, Schachner HC, Bailey T (2013). Evaluation of a combined blood glucose monitoring and gaming system (Didget) for motivation in children, adolescents, and young adults with type 1 diabetes. Pediatr Diabetes.

[20] Thompson D, Baranowski T, Buday R (2010). Conceptual model for the design of a serious video game promoting self-management among youth with type 1 diabetes. J Diabetes Sci Technol.

[21] Kharrazi H (2009). Improving healthy behaviors in type 1 diabetic patients by interactive frameworks. AMIA Annu Symp Proc.

[22] Ebrahimpour F, Najafi M, Sadeghi N (2014). The design and development of a computer game on insulin injection. Electron Physician.

[23] Thompson D (2014). Talk to me, please! The importance of qualitative research to games for health. Games Health J.

[24] Fox MP (2009). A systematic review of the literature reporting on studies that examined the impact of interactive, computer-based patient education programs. Patient Educ Couns.

[25] Ahola AJ, Groop PH (2013). Barriers to self-management of diabetes. Diabet Med.

[26] Fails JA (2013). Methods and Techniques for Involving Children in the Design of New Technology for Children. FNT in Human–Computer Interaction.

[27] Fico G, Fioravanti A, Arredondo MT, Leuteritz J, Guillén A, Fernandez D (2011). A user centered design approach for patient interfaces to a diabetes IT platform. Conf Proc IEEE Eng Med Biol Soc.

[28] Bee H (2003). The Developing Child, 9th Edition.

[29] Elo S, Kyngäs H (2008). The qualitative content analysis process. J Adv Nurs.

[30] Elo S, Kaariainen M, Kanste O, Polkki T, Utriainen K, Kyngas H (2014). Qualitative content analysis: a focus on trustworthiness. SAGE Open.

[31] Streisand R, Monaghan M (2014). Young children with type 1 diabetes: challenges, research, and future directions. Curr Diab Rep.

[32] Siminerio L, Albanese-O'Neill A, Chiang JL, Hathaway K, Jackson C, Weissberg-Benchell J, Wright JL, Yatvin AL, Deeb LC, American Diabetes Association (2014). Care of young children with diabetes in the child care setting: a position statement of the American Diabetes Association. Diabetes Care.

[33] Moreira PL, Dupas G (2006). [Vivendo com o diabetes: a experiencia contada pela crianca]. Rev Latino-am Enfermagem.

[34] Roper SO, Call A, Leishman J, Ratcliffe GC, Mandleco BL, Dyches TT, Marshall ES (2009). Type 1 diabetes: children and adolescents' knowledge and questions. J Adv Nurs.

[35] Wiley J, Westbrook M, Long J, Greenfield JR, Day RO, Braithwaite J (2014). Diabetes education: the experiences of young adults with type 1 diabetes. Diabetes Ther.

[36] Nansel TR, Haynie DL, Lipsky LM, Wang J, Mehta SN, Laffel LM (2013). Relationships among parent and youth healthful eating attitudes and youth dietary intake in a cross-sectional study of youth with type 1 diabetes. Int J Behav Nutr Phys Act.

[37] American Association of Diabetes Educators Teaching injection technique to people with diabetes.

[38] Cunningham MT, Malachi M (2013). Lipohypertrophy in insulin-treated diabetes: prevalence and associated risk factors. J Diabetes Nurs.

[39] Fu AZ, Qiu Y, Radican L (2009). Impact of fear of insulin or fear of injection on treatment outcomes of patients with diabetes. Curr Med Res Opin.

[40] Rzeszut JR (2011). Children with diabetes: the impact of fear of needles. J Pediatr Nurs.

[41] Floch JL, Bauduceau B, Lévy M, Mosnier-Pudar H, Sachon C, Kakou B (2008). Self-monitoring of blood glucose, cutaneous finger injury, and sensory loss in diabetic patients. Diabetes Care.

[42] Rewers M, Pihoker C, Hanas R, Swift P, Klingensmith GJ (2014). Assessment and monitoring of glycemic control in children and adolescents with diabetes. Pediatr Diabetes.

[43] Smart CE, Annan F, Bruno LP, Higgins LA, Acerini CL, International Society for Pediatric and Adolescent Diabetes (2014). ISPAD Clinical Practice Consensus Guidelines 2014. Nutritional management in children and adolescents with diabetes. Pediatr Diabetes.

[44] Samson J (2006). Exploring young people's perceptions of living with type 1 diabetes. J Diabetes Nurs.

[45] Baranowski T, Beltran A, Chen T, Thompson D, O'Connor T, Hughes S, Diep C, Baranowski JC (2015). Predicting use of ineffective vegetable parenting practices with the model of goal directed behavior. Public Health Nutr.

[46] Beltran A, O'Connor T, Hughes S, Baranowski J, Nicklas TA, Thompson D, Baranowski T (2012). Alpha test of a videogame to increase children's vegetable consumption. Games Health J.

[47] Robertson K, Riddell MC, Guinhouya BC, Adolfsson P, Hanas R (2014). ISPAD Clinical Practice Consensus Guidelines 2014. Exercise in children and adolescents with diabetes. Pediatr Diabetes.

[48] Fowler MJ (2008). Hypoglycemia. Clin Diabetes.

[49] Brennan PF, Fink SV (1997). Health Promotion and Interactive Technology: Theoretical Applications and Future Directions.

[50] Kato PM, Cole SW, Bradlyn AS, Pollock BH (2008). A video game improves behavioral outcomes in adolescents and young adults with cancer: a randomized trial. Pediatrics.

[51] Baranowski T, Baranowski J, Thompson D, Buday R, Jago R, Griffith MJ, Islam N, Nguyen N, Watson KB (2011). Video game play, child diet, and physical activity behavior change a randomized clinical trial. Am J Prev Med.

